# A novel mitochondrial DNA deletion in a patient with Kearns-Sayre syndrome: a late-onset of the fatal cardiac conduction deficit and cardiomyopathy accompanying long-term rGH treatment

**DOI:** 10.1186/1471-2431-13-27

**Published:** 2013-02-20

**Authors:** Monika Obara-Moszynska, Jaroslaw Maceluch, Waldemar Bobkowski, Artur Baszko, Oskar Jaremba, Maciej R Krawczynski, Marek Niedziela

**Affiliations:** 1 Department of Pediatric Endocrinology and Rheumatology, Poznan University of Medical Sciences, 27/33 Szpitalna Street, 60-572, Poznan, Poland; 2 Department of Pediatric Cardiology and Nephrology, Poznan University of Medical Sciences, Poznan, Poland; 3 2nd Department of Cardiology, Poznan University of Medical Sciences, Poznan, Poland; 4 Department of Medical Genetics, Poznan University of Medical Sciences, Poznan, Poland

**Keywords:** Growth hormone treatment, Mitochondrial disease, Atrio-ventricular block, Cardiomyopathy, Short stature

## Abstract

**Background:**

Kearns-Sayre Syndrome (KSS) is a multisystem disorder caused by a dysfunction of the oxidative phosphorylation system within mitochondria. Mitochondrial DNA (mtDNA) rearrangements are a key molecular feature of this disease, which manifest a broad phenotypic spectrum.

**Case presentation:**

Here, we present a boy with KSS whose symptoms included cardiac conduction deficit, cardiomyopathy and growth hormone (GH) deficiency. The patient showed typical symptoms for KSS from early childhood (chronic progressive external ophthalmoplegia, retinopathy, short stature). Long-range PCR analysis disclosed a 7663-base pair heteroplasmic deletion in the mtDNA encompassing nucleotides 6340–14003. At 12 years of age, GH deficiency was recognized and recombinant growth hormone (rGH) therapy was started. At 15 years of age, a complete atrioventicular block was diagnosed and the patient received a pacemaker. During the following 6 months, progressive deterioration of the left ventricle was observed and an echocardiogram showed features of dilated cardiomyopathy. The rGH treatment was then discontinued at a final height of 163 cm. Unfortunately, due to multi-organ insufficiency and inflammation, the patient died at the age of 18 years.

**Conclusions:**

The response to rGH therapy in the patient was very satisfactory. The large mtDNA deletion had no apparent impact on the response to rGH. Cardiac disturbances occurred as part of the syndrome and were not related to rGH therapy; however, the progression of the disease led to death.

## Background

Kearns-Sayre Syndrome (KSS) is a rare metabolic disorder which belongs to the group of mitochondrial cytopathies [1]. The diagnosis of KSS is made based on the classic triad of symptoms: onset of the disease before 20 years of age, progressive external ophthalmoplegia (PEO) and pigmentary retinopathy (PR) [2]. KSS is manifested also by other systemic abnormalities: cardiac conduction defects, different neurological abnormalities and several endocrine disorders [3]. Progression of the clinical symptoms in KSS usually lead to death before the age of 30. Spontaneous mitochondrial DNA (mtDNA) rearrangements underlie this disease. A variety of deletions and/or duplications in mtDNA, affecting genes encoding respiratory chain proteins, are found in most cases [4-6]. This leads to defects in energy production by mitochondria and dysfunction of many tissues. This is particularly noticeable in tissues with a high energy demand - like muscles and the brain. The mutated mtDNA coexists with normal molecules (heteroplasmy) and the proportion of mutated to normal mtDNA correlates with the severity of clinical symptoms. There is no effective treatment for KSS and the complicated character of clinical features can impede the establishment of the correct diagnosis [1].

In this report we present the comprehensive clinical diagnosis of KSS in a young male. The patient was initially hospitalized based on neurological symptoms, followed by a diagnosis of his endocrinopathies. The most serious features of the disease - a cardiac conduction deficit and cardiomyopathy developed some years later. The final diagnosis was made several years after the first symptoms appeared, and was established using molecular biology techniques.

## Case presentation

The presented patient is an 18-year-old boy with Kearns-Sayre Syndrome coexisting with GH deficiency, complicated by a cardiac conduction deficit and cardiomyopathy.

**Figure 1:**
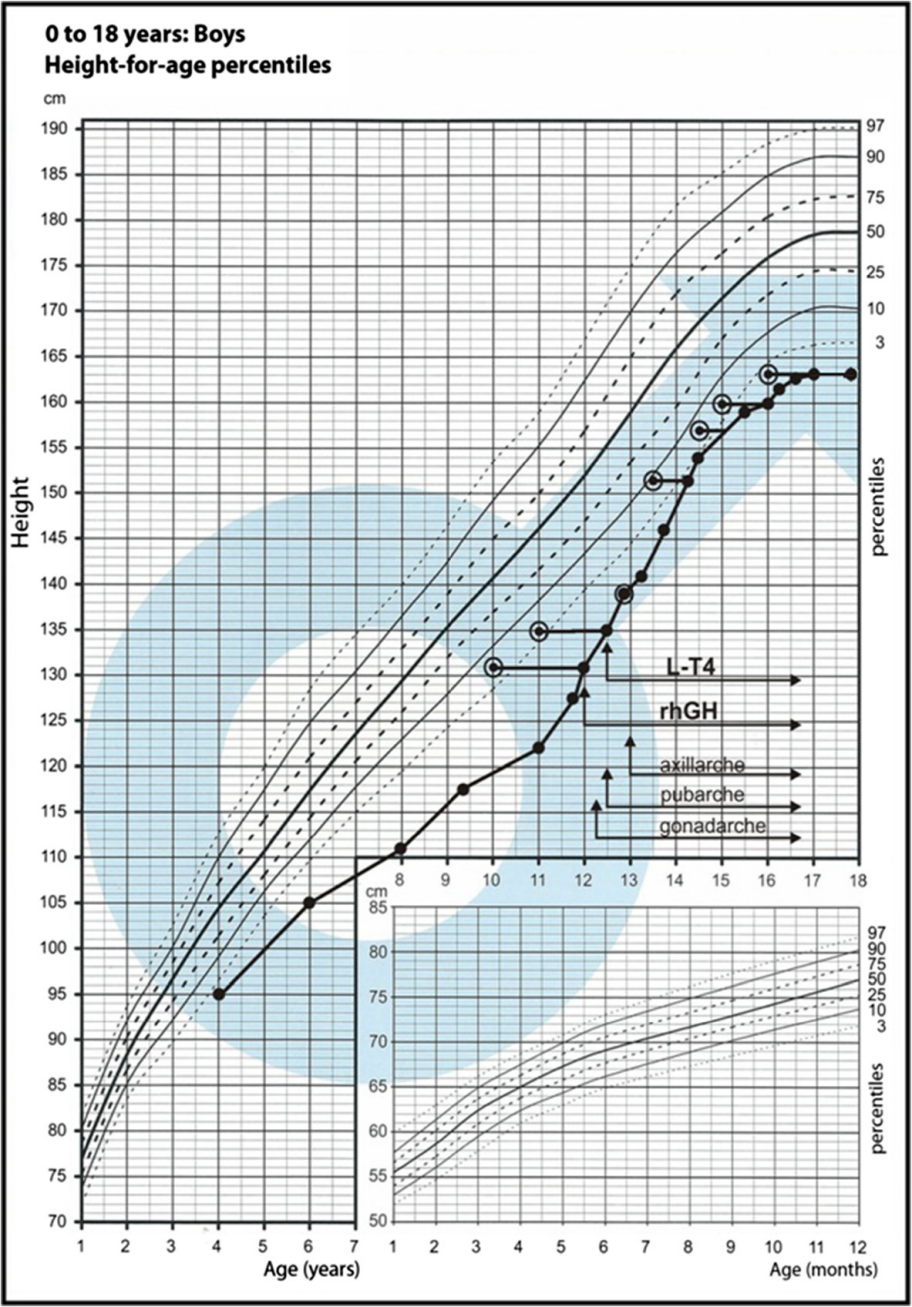
Growth chart with patient growth pattern.

**Figure 2:**
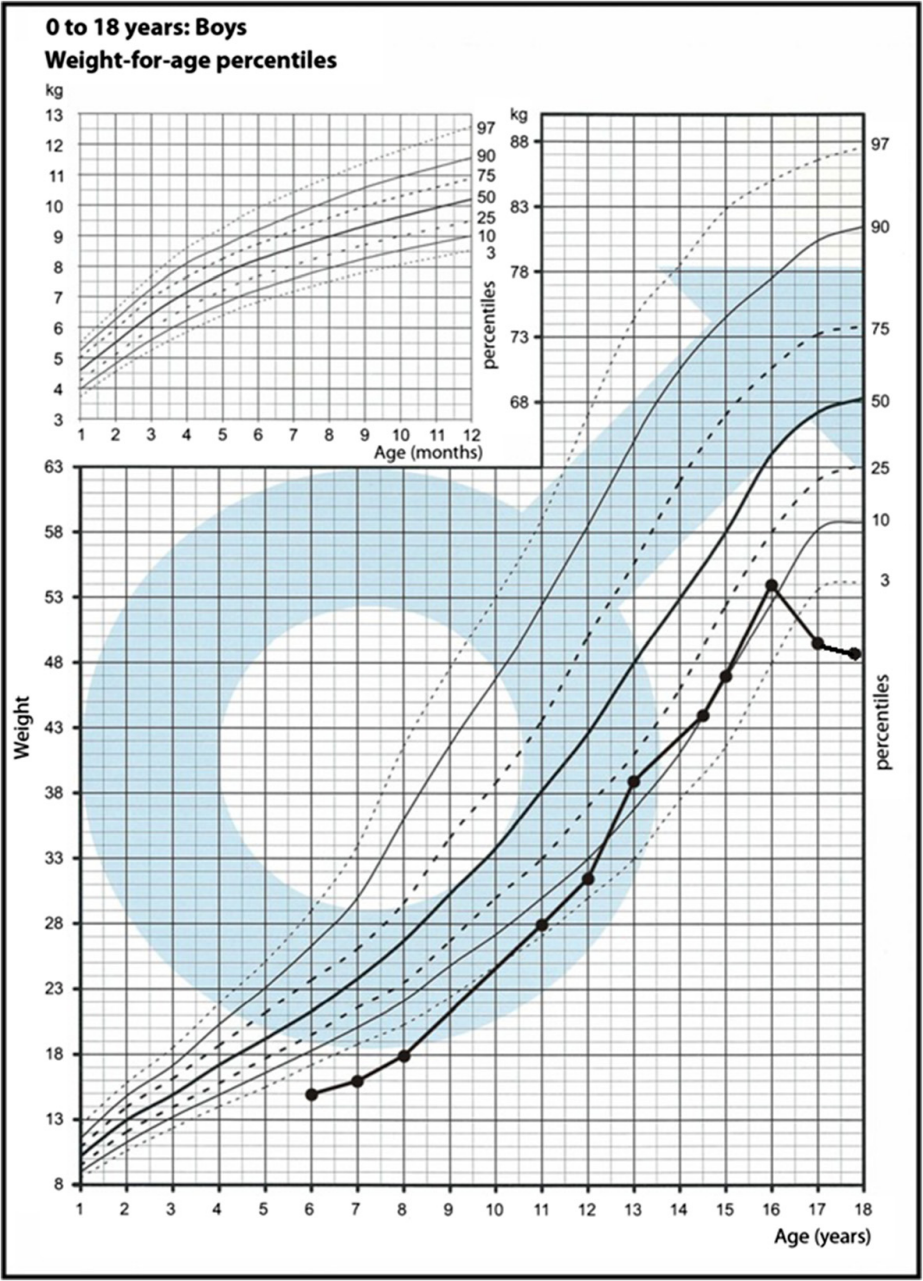
Growth chart with patient weight pattern.

The child was adopted in early childhood and the family history is unknown. The delivery was at term and uncomplicated. The boy was born small for gestational age with a birth weight of 2500 g (−2,43 SD) and Apgar score of 10 points. From the second year of life, chronic progressive external ophthalmoplegia (CPEO) was observed. It started with unilateral ptosis (left eye), which gradually progressed to bilateral at 11 years of age. Additionally, extra-ocular muscle palsy and pigmentary retinopathy (PR) were observed. From early childhood, the boy displayed short stature by relative normal body weight (Figure 1, Figure 2).

At the age of 11, he was admitted to the neurology clinic. An electromyogram (EMG) was performed and revealed a myogenic pattern. A magnetic resonance imaging (MRI) of the head showed hypoplasia of the pituitary gland. Examination of cerebrospinal fluid, auditory evoked potentials of the brainstem and electroencephalogram (EEG) were all normal. Psychological examination identified an IQ at average level. The genetic analysis showed a normal male (46,XY) karyotype.

Due to the clinical picture (CPEO, PR) and EMG examination the diagnosis of Kearns-Sayre Syndrome (KSS) was proposed.

Further endocrine work-up detected a partial growth hormone deficiency. At 12 years of age, recombinant growth hormone (rGH) therapy was started with a standard dose 0.025 mg/kg/day (Table 1). During rGH treatment, the patient developed secondary hypothyroidism.

**Table 1:** Selected parameters during rGH therapy

	**Age [years]**	**Height SDS**	**Bone age [G&P] [years]**	**Height velocity [cm/year]**	**Predicted adult height based on Bayley-Pinneau method [cm]**	**rGH Dose [mg/kg/24 h]**
the beginning of rGH therapy	12	−3.5	10	8.5	161.9	0.024
after 6 months of rGH therapy	12 6/12	−2.9	11	10.5	164.6	0.024
after 12 months of rGH therapy	13	−2.6	13	7.5	161.7	0.024
after 2 years of rGH therapy	14 3/12	−2.2	13 6/12	10.0	168.5	0.022
after 3 years of rGH therapy	15 2/12	−2.3	14 6/12	4.5	168.5	0.018
after 4 years of rGH therapy	16	−2.3	15	2.5	166.5	0.018
after 5 years of rGH therapy	17	−2.6	16	1.3	166	Discontinuation of treatment

The onset of puberty was spontaneous and initiated at 12 years and 3 months of age.

HbA1c levels were borderline when rGH treatment was started and also 2 years later- they were 5.9% and 6.0% respectively (Table 2).

**Table 2:** Levels of hormones and other biochemical parameters in blood samples (*abnormal values are given in bold*)

**Laboratory tests**	**10 yrs**	**12**	**14**	**16**	**Reference range**
**Results**					
TSH (μIU/ml)	2.1	2.99	**0.44**	**0.36**	0.470-4.640
fT4 (ng/dl)		**0.63**	1.04	1.14	0.71-1.85
ACTH (pg/ml)	31.7			30.7	10-60
Cortisol (ng/ml)				143	94-260
LH (mIU/ml)				7.6	2.0-12.0
FSH (mIU/ml)				6.3	1.0-8.0
Testosterone (nmol/l)				19.83	8.84-26.1
Prolactin (ng/ml)				7.09	3.24-29.12
IGF-1 (ng/ml)	212	428	511	602	
max GH (after clonidine) (ng/ml)	**4.3**				>10
max GH (after insulin) (ng/ml)	**1.5**				>10
HbA1c (%)		5.9	6.0	5.3	<6.1
Glucose (mg/dl)	91	88	84	79	59-101
anti-GAD (U/ml)			0.2	0.2	<1
IAA (%)			4.3	5.4	<5.5
IA-2 (U/ml)			0.1		<1
Na^+^ (mmol/l)	139		138	144	132-145
K^+^ (mmol/l)	3.44		3.39	4.97	3.1-5.1
Ca (mmol/l)	2.55		2.61	2.45	2.1-2.6
Mg (mg/l)	**17.2**		**14.3**	23.1	18.2-23.1
P (mg/dl)			4.52	4.63	4.50-5.52
AspAt (U/l)			25	23	1-40
AlAt (U/l)			20	23	1-45
Creatinine (mg/dl)			0.53	**1.43**	0.6-1.3
Total protein (g/dl)			7.6		6.0-8.0
IgA tTG				neg	
Cholesterol			192	121	110-230

Further analysis revealed hyposecretion of insulin and an elevated blood glucose in oral glucose tolerance test (OGTT), glutamic acid decarboxylase (GAD), anti-insulin (IAA) and insulinoma antigen 2 (IA2) antibodies were all tested and were negative. Long acting insulin therapy was started and rGH dose was reduced. The glucose concentration then normalized. Insulin-like growth factor 1 (IGF-1) levels during rGH administration were within the normal range, however, usually higher than the mean expected for age and sex. Levels of hormones and other biochemical parameters in blood samples are shown in Table 2.

**Figure 3:**
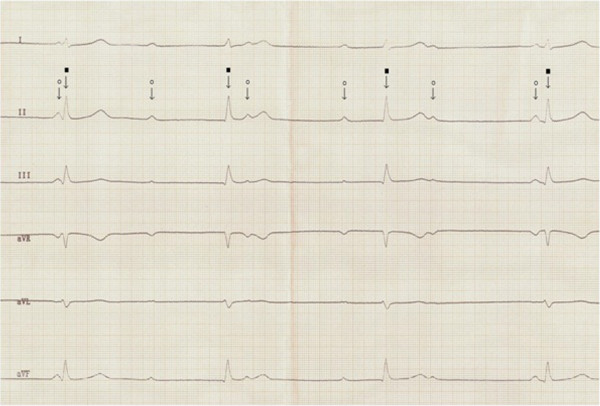
**Standard ECG showing complete atrio-ventricular block.** Atrial rhythm (white circle) of 70 bpm and ventricular escape rhythm of 42 bpm, narrow QRS (black square). Paper speed 50 mm/s.

At 15 years of age, during a physical examination bradycardia was noticed. The patient displayed no major complaints concerning the cardiovascular system and his vital signs were all within normal limits - except for bradycardia. A detailed diagnostic work-up was ordered to determine the cause of the bradycardia. An electrocardiogram (ECG) examination was also performed and confirmed an intracardiac conduction abnormality characteristic of complete atrio-ventricular block with slow regular ventricular escape rhythm (rate of 35–38 bpm) with wide QRS complexes (Figure 3). The rate of the atrial rhythm was 80 bpm. As a next step, 24 h Holter monitoring was performed which confirmed the presence of a complete heart block, with minimum resting heart rate of 32 bpm, maximum of 49 bpm and an average of 39 bpm. Echocardiogram examination was remarkable for mitral and tricuspid insufficiencies - both of second degree. The calculated shortening fraction was 36%, with a left ventricular ejection fraction value of 65%, good contractility was observed and the dimensions of the heart chambers were within normal limits. The next step performed was a surgical implantation of an artificial biventricular pacemaker under general anesthesia. The first active electrode was inserted through the basilic vein to the right ventricle and eventually placed in the septal part of right ventricular outflow tract, whereas the second active electrode was inserted through the subclavian vein and placed in the right atrium. The dual-chamber pacemaker IDENTITY ADxXL DR from St. Jude Medical was successfully installed and programmed. Control electrocardiogram revealed a pacemaker rhythm of 75 bpm with ventricular stimulation controlled by the native sinus rhythm.

During the next 6 months, a progressive deterioration of the left ventricle was observed. In the performed echocardiogram, features of dilated cardiomyopathy were revealed. The picture of the heart suggested left ventricle noncompaction (LVNC), which is a congenital genetic developmental defect of heart muscle. At that time the boy had an implanted cardioverter – defibrillator with resynchronising stimulation. The prognosis was at that time also uncertain.

At the age of 17, the rGH therapy was discontinued due to a deceleration of height velocity - with a final obtained height of 163 cm. Unfortunately, due to multi-organ insufficiency and inflammation, the patient died at the age of 18 years.

**Figure 4:**
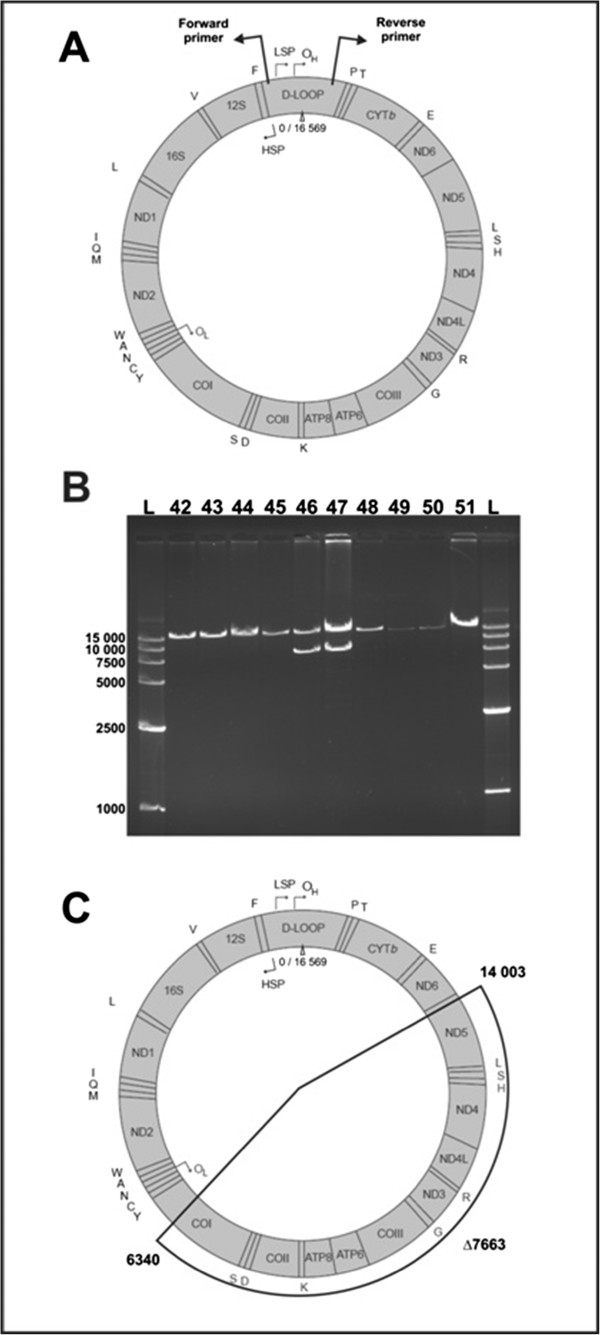
**A,B,C.** Results of molecular analysis of patient’s mitochondrial DNA. **A** - Schematic localization of PCR primers used for the detection of deletion in mtDNA; **B** - Result of Long-Range PCR detection of mitochondrial deletion, numbers represent samples from different patients suspected for KSS, numbers 46 and 47 represent two samples from presented patient, L indicates molecular weight ladder (GelPilot High Range Ladder, QIAGEN); **C** - Schematic localization of detected mtDNA deletion.

Molecular analysis of mitochondrial DNA was performed with total DNA isolated from the patient’s peripheral blood. For molecular analysis, we obtained two separate blood samples collected at a two year interval. This allowed us to compare how genetic disturbances changed, if they did at all, with the progression of disease. 20 ng of DNA was used for amplification of specific PCR products representing almost the whole mtDNA molecule (Figure 4A). Control reactions were performed using a DNA template from a healthy individual. Additional, smaller PCR products after gel electrophoresis were observed that indicated the presence of a deletion (Figure 4B). The analysis of sequences of the small product mapped the deletion to the region between nucleotides 6340 and 14 003 on the mtDNA molecule (Figure 4C). This big deletion (7663 bp) removes almost half of the mtDNA molecule along with genes which encode critical proteins in the mitochondrial electron transport chain: cytochrome c oxydase, NADH dehydrogenase, ATP synthase and 8 tRNAs. Such a dramatic rearrangement of mitochondrial DNA must have a significant impact on mitochondrial function, but the influence of this deletion on clinical symptoms and the severity of the disease also depends on the level of heteroplasmy.

Quantitative analysis of the heteroplasmy level was performed by Real-Time PCR technique. 20 ng of total DNA was analyzed using QuantiTect SYBR Green PCR Kit (Qiagen) on a Mastercycler ep realplex machine (Eppendorf) and four specially designed primer sets. All reactions were done in triplicate. Melting curve analysis was added at the end of all reactions. Relative expression ratios of mtDNA levels were calculated using the ΔC_T_ method. As a control of the total mtDNA level, we analyzed the DNA of five healthy individuals, using the same primers as for the patient. Amplification results were presented as a relative expression towards the expression level of a housekeeping, nuclear gene - GAPDH. The results of this analysis revealed that the total level of mtDNA in the patient’s blood was comparable to the normal mtDNA level in the first, older DNA sample, but significantly higher (2.6×) in the second sample.

Next, we tested the heteroplasmy level, analyzing in separate reactions only normal, non-deleted mtDNA molecules, or only molecules with the deletion. A comparison of the results of these two reactions revealed that mtDNA molecules with the deletion comprise about 3% of the total mtDNA pool in the patient’s blood.

**Figure 5:**
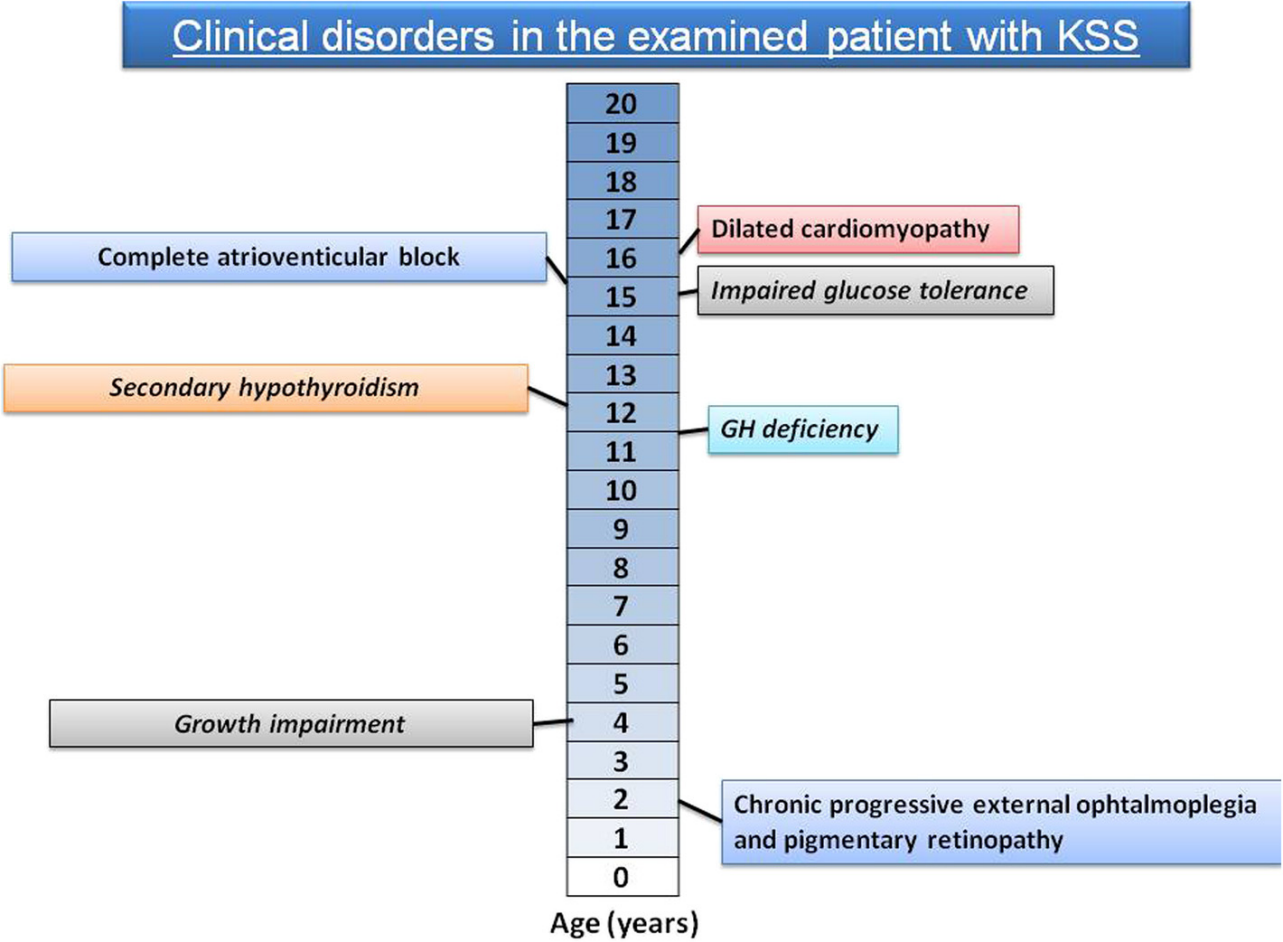
Chronologic occurrence of clinical symptoms in presented boy.

The presented patient is a case with a typical symptom sequence of KSS (Figure 5) and with a big, novel deletion in mtDNA.

This observation, with changing total mtDNA, fits well with the standard pattern of genetic rearrangements during the course of mitochondrial cytopathies. It was demonstrated that in patients with single deletions at high heteroplasmy levels the amount of mitochondria within the cells was nearly doubled [7]. Therefore, even considerably reduced levels of enzyme activity can be compensated for by increased amounts of mitochondria. In another investigation, an approximately nine-fold amplification of mtDNA in muscle was detected [8]. Other reports also indicate a synchronous increase in the levels of deleted and normal mtDNA in the course of KSS [9].

The low percentage of the deleted form of mtDNA compared to total mtDNA was not surprising, because the level of mutated mtDNA in blood is usually very low and sometimes undetectable [5]. The observed level of mutated mtDNA in blood lymphocytes does not correlate with the severity of observed clinical symptoms of the disease. It is also likely that the level of mutated mtDNA in other tissues was much higher, but that was impossible for us to test because we lacked a muscle biopsy specimen for analysis.

The boy had the classic triad, plus the cardiac conduction defect. The dominant disturbances at the beginning of disease manifestation were ophthalmologic, then endocrinologic. With the development of the disease, the cardiac complications became very severe, subsequently leading to a life-threatening state.

Most cases of KSS (like the presented patient) and other mitochondrial cytopathies are first diagnosed as various endocrine disorders and the diagnosis of KSS is later established after several years [10,11].

Endocrinologic pathologies in the presented patient were as follows: GH deficiency, hypothyroidism and impaired endocrine function of the pancreas. At the beginning of rGH treatment the prognosis concerning the therapy response was uncertain. The improvement of height velocity during rGH therapy was very satisfactory, and exceptional if compared to other children treated with KSS in our clinic. After glucose metabolism became impaired and the dose of rGH was reduced, the patient still accelerated on his growth chart. This means that the growth signal was transduced properly, despite the main disease. The large mtDNA deletion and resultant metabolic defects had no impact on treatment response to rGH.

The cardiac manifestations of KSS are the most important aspects of the disease for determining the prognosis [12]. Manifestations of cardiac disease occur in 57% of patients with Kearns-Sayre Syndrome, including syncopal attacks, heart failure and cardiac arrest [13]. Patients with KSS typically develop cardiac conduction defects that progress to complete heart block and then manifest clinically as heart failure, syncope or sudden death. Cases described in the literature show the progressive course of life-threatening conduction abnormalities in KSS [14]. Patients who do not have conduction disturbances should be closely monitored for the development of conduction defects. In all patients with mtDNA mutations, not just KSS patients, mortality among those with no cardiac disease is 26% vs. 71% in patients with cardiomyopathy [15]. Pacemakers are recommended for all patients with neuromuscular diseases (including KSS) who have developed atrioventricular (AV) block, however, prophylactic pacemaker placement prior to the development of third-degree or advanced second-degree AV block is discussed with patients and families as well [16]. Based on the International Society of Heart and Lung Transplantation database, 6 patients who received heart transplantation between 1990 and 2003 had a diagnosis of myopathy due to a mitochondrial pathology [17].

The question is whether rGH treatment could have an impact on the cardiological complications in the patient. GH, both in excess and in deficient states can be associated with increased cardiovascular disturbances. Exposure to high GH levels causes the hypertrophy of cardiac myocyte [18]. Patients with GH oversecretion develop acromegalic cardiomyopathy, biventricular hypertrophy and eventual heart failure [19]. Dysrhythmias are also observed in acromegaly. Most common are ventricular arhythmias, like premature ventricular beats, ventricular bigeminy and ventricular tachycardia [20]. In GH deficiency an increased risk of ischemic heart disease is described [21]. GH deficiency results in a change in body composition, bone mineral density, an altered lipid profile, and decreased muscle mass. Therefore the proper balance within the GH and IGF-1 axis is necessary for maintaining normal cardiac function.

The presented patient had growth hormone deficiency and was treated with substitution doses of rGH. The IGF-1 concentration during rGH administration was within the normal range throughout the entire period of treatment. Therefore, the proper homeostasis of GH and IGF-1 axis was maintained. There is no evidence that rGH could have had a negative impact on cardiac function in this boy. It is rather to be suspected that treatment with rGH could even have a beneficial influence on the heart. There are some studies that show a positive influence of rGH on myocardium, also in dilated cardiomyopathy. GH can improve left ventricle structure and cardiac output, leading to a better hemodynamic profile of the heart [22,23].

## Conclusions

The presented case shows the classical clinical characteristics of Kearns-Sayre Syndrome. The urgent and rapid manifestation of severe cardiac dysfunction emphasizes the necessity of careful cardiological follow-up in KSS. The response to rGH in KSS is somewhat unpredictable, similarly to cardiac dysfunction, and there is no proof that rGH treatment contributes to the onset of the conduction block.

## Consent

Written informed consent was obtained from the mother of the patient for publication of this case report and any accompanying images. A copy of the written consent is available for review by the Series Editor of this journal.

## Abbreviations

KSS: Kearns-Sayre Syndrome; mtDNA: Mitochondrial DNA; GH: Growth hormone; rGH: Recombinant growth hormone; PEO: Progressive external ophthalmoplegia; PR: Pigmentary retinopathy; CPEO: Chronic progressive external ophthalmoplegia; EMG: Electromyogram; MRI: Magnetic resonance imaging; EEG: Electroencephalogram; OGTT: Oral glucose tolerance test; GAD: Glutamic acid decarboxylase; IAA: Anti-insulin antibodies; IA2: Insulinoma antigen 2; IGF-1: Insulin-like growth factor 1; ECG: Electrocardiogram; PCR: Polymerase chain reaction

## Competing interests

None of the authors of the manuscript has declared any financial and non-financial competing interests in relation to this manuscript.

## Authors’ contributions

MO-M conceptualized and designed the study, drafted the initial manuscript and approved the final manuscript as submitted. JM was responsible for molecular analysis, drafted the initial manuscript, completed the molecular analysis in the manuscript and approved the final manuscript as submitted. WB was involved in the cardiologic care of the patient, coordinated and supervised the cardiologic part of the manuscript and approved the final manuscript as submitted. AB was involved in the cardiologic care of the patient, approved the final manuscript as submitted. OJ was involved in the cardiologic care of the patient, was involved in the cardiologic part of the manuscript and approved the final manuscript as submitted. MRK was involved in the molecular part of the study, coordinated and supervised the genetic part of the manuscript, approved the final manuscript as submitted. MN conceptualized and designed the study, coordinated and supervised the study, drafted the initial manuscript and approved the final manuscript as submitted.

## Pre-publication history

The pre-publication history for this paper can be accessed here:

http://www.biomedcentral.com/1471-2431/13/27/prepub
